# Physical Activity, Mental Health, and Wellbeing among Older Adults in South and Southeast Asia: A Scoping Review

**DOI:** 10.1155/2019/6752182

**Published:** 2019-11-17

**Authors:** Shanti Kadariya, Rupesh Gautam, Arja R. Aro

**Affiliations:** ^1^Unit for Health Promotion Research, University of Southern Denmark, Esbjerg, 6700, Denmark; ^2^School of Public Health and Social Work, Queensland University of Technology, Kelvin Grove 4059, Brisbane, Australia

## Abstract

**Background:**

Physical activity is believed to enhance body functions and sense of wellbeing in general population.

**Objectives:**

This study aimed to explore physical activity measures; and the association between those measures, and mental wellbeing among older adults in South and Southeast Asia.

**Methods:**

A systematic search was made in CINHAL, EMBASE, PubMed, and PsycINFO. Articles published between 2008 and 2018 were selected with participants aged 60 years and above, living at home, community, supported housing, or residential care homes, with no diagnosed/limiting illness.

**Results:**

Five observational and four interventional studies on physical activity were analysed. Depression and sleep quality were the commonest outcome variables. Exercise frequency, regularity, and duration were found to positively impact mental wellbeing.

**Conclusion:**

Physical activity was generally found protecting against depression and improved sleep quality of older adults from South and Southeast Asia. Future studies should focus on more objective measures of physical activity.

## 1. Introduction

### 1.1. Background

Ageing is a chronic condition of human life cycle and is accompanied by conditions such as cardiovascular disease, hypertension, diabetes mellitus, cancer, and/or mental illness [1].

Ageing is a long-term result of accumulation of several kinds of molecular and cellular damage over time. This stage in the life cycle is marked by a gradual decline in physical and mental capacity, increased risk of disease and finally death. Ageing is not just biological but also has a social facet to it marked by retirement, relocation to new living arrangements as well as deaths of partners, friends, and relatives [2].

Globally, the population is ageing rapidly, and it is estimated that older people or elderly, defined as those aged 60 years and over, will constitute 22% of the total world population by 2025 [3]. Older people have limited regenerative activities but are more prone to the increase in age-related diseases and disabilities, with several social and financial implications to the individual themselves and to the society. Furthermore, mental health issues constitute a critical aspect of health problems in older adults. It is estimated that more than 20% of older adults aged 60+ years suffer from mental and neurological disorders globally. Mental and neurological disorders account for 6.6% of the total disability (DALYs) for this age group [4].

Due to several reasons like lack of independence, frailty, illness, separation, isolation, and simply due to their age, among other reasons, older people are at disproportionately higher risk of suffering from mental health problems. What is further noteworthy for all age groups, but especially so for the older adults, is that physical health has an impact on mental health just like mental health has an impact on physical health [4]. Therefore, along with a continued focus on extending their life expectancy, it is also necessary to understand the strategies to maintain sound mental health and wellbeing in older adults [5].

Physical activity refers to any bodily movement produced by skeletal muscles that needs energy as an input. It includes all daily activities like playing, carrying out household chores, travelling, and activities during work and recreational pursuits [6].

Participation in light and moderate physical activities have been known to be effective on delaying the functional decline among older adults, thereby promoting healthy ageing in them [7]. Different studies have assessed the relationship between active participation in exercise and sports and elderly wellbeing. Many of those studies found a positive correlation between exercise frequency, wellbeing, and body function improvement among older people. As a result, there is a level of understanding that regular participation in exercise habits enhances body functions and improves their sense of wellbeing. Some other benefits found by these studies are improvement in body coordination, flexibility, better sleep quality, decrease in depression and anxiety; and improvement in overall psychological wellbeing [8–10].

Mental health services in Southeast Asian countries have been reported as inadequate, and suffering from issues like lack of proper funding, lack of health workers and advocacy groups, urban-concentration of service providers with poor quality, and unaffordable services [11]. As such, the provision of mental health services in these countries is understood to be limited. With such inadequacies in mental health service provision for the general population, it is hard to expect a specific service focus on the mental health of the elderly population.

The state of mental health service provision in South Asia is not very promising either. Factors like large scale natural disasters and conflicts within the region have fuelled the poor state of mental health in South Asian population. Depression, anxiety, and posttraumatic stress disorders have been found to be some of the commonly occurring mental conditions, particularly resulting from conflicts. Apart from conflicts, other factors like female sex, displacement, being widowed/divorced, low income and/or education and food insecurity have been identified as some of the reasons responsible for poor mental health status in this region. Old age is another reason cited to be responsible for the current state of mental health in South Asia, which is beginning to experience an unprecedented increase in the proportion of elderly population. Furthermore, lack of financing, lack of prioritization of mental health, stigma associated with mental health disorders, inadequate specialist health workers, and lack of integration with primary health care have been identified as some of the systemic reasons that have contributed to poor mental health service provision in the region [12].

### 1.2. Rationale

In the pretext of lack of priority, inadequate service provision and lack of specific interventions targeting the old age groups, focus on primordial and primary preventive measures could prove helpful in countries with rising older adult populations and limited resources. Studies on effectiveness of physical activity on mental health and wellbeing of senior adults have, to a great extent, been conducted in western populations or within western contexts. Due to their different socioeconomic contexts and cultures of work, retirement, and leisure than the western countries, having an understanding of such association between physical activity, mental health, and wellbeing among older people living in South and Southeast Asia is important. Although in limited number and focused on some specific countries of the region, studies have been conducted in the area of physical activity, mental health, and wellbeing among senior adults in South and Southeast Asia. However, attempts have not yet been made to gather the scattered information in one study. Therefore, we aimed to conduct a scoping review of the studies published in this research domain in this geographic area to generate an aggregated knowledge of all these studies and also to inform future studies.

### 1.3. Objectives

In order to map the literature, identify key concepts already explored and the existing gap in knowledge from South and Southeast Asia on physical activity and its influence on mental health and wellbeing among senior adults from the region, this study was conducted as a scoping review. Through a narrative overview, this study aimed to meet the following major objectives:

Gather information on the types of physical activities and/or exercise measures studied in South and Southeast Asia in their relationship with mental health and wellbeing among older adults.

Understand the association between physical activity and/or exercise practices, mental health, and wellbeing among older adults in South and Southeast Asia.

Understand the effect of physical activity and/or exercise interventions on mental health and wellbeing among senior adults in South and Southeast Asia.

## 2. Materials and Methods

This study followed the 5 stages of York framework outlined by Arksey and O'Malley, [13] and PRISMA Extension for Scoping Reviews (PRISMA-ScR) [14].

### 2.1. Identifying the Research Questions

We decided on the research questions as our guide for article search. The questions helped us identify the general characteristics of the study in terms of person, place, and time; different physical activities measured in everyday lives as well as those introduced as interventions, different forms of mental health outcome variables chosen by studies and the ways the results were presented. Our questions were as follows:

(i) What is known about physical activity and/or exercise, mental health, and wellbeing among older adults in South and Southeast Asia?
(ii) What different forms of physical activity and/or exercise practices have been studied with an aim to understanding their influence on mental health and wellbeing of older adults in South and Southeast Asia?
(iii) What is known about the association between those different forms of physical activity and/or exercise practices (observations as well as interventions), mental health, and wellbeing among senior adults in South and Southeast Asia?

### 2.2. Identifying Studies Relevant to the Research Questions

In order to decide on the studies to be included in the review, we defined some inclusion and exclusion criteria before beginning our literature search. Once we had developed our search criteria, we decided on the databases to search to look for the articles.

### 2.3. Inclusion Criteria

Studies with participants aged 60 years and over; living at home, in the community, in supported housing or in residential care homes; with no diagnosed illness/limiting illness; and who were able to walk without assistance were selected. Only those studies which assessed mental health and wellbeing of older adults in relation to physical activity were included. We limited our search to only those studies that included older adults with no diagnosed illness or limiting illness because we wanted to understand how physical activity influences supposedly healthy older adults' mental health and wellbeing. While exploring this research question in older adults with one or multiple morbidities is equally important, it is outside the scope of this study. Mental wellbeing, for the purpose of this study was defined to include better sleep, reduced depression and anxiety, and/or similar other pyschological outcomes. We only reviewed studies conducted in the last 10 years in order to incorporate relatively recent findings. Therefore, observational or experimental studies conducted between 2008 and 2018 in South Asian and Southeast Asian countries targeted at older adults aged 60 years and above [according to the classification of Asian Development Bank (ADB) [15] and South Asian Association for Regional Cooperation (SAARC)] [16] were included.

### 2.4. Exclusion Criteria

The studies were excluded if they considered older adults undergoing (a) treatment for a clinically diagnosed physical or mental illness, (b) assessment(s) for long-term continuing care, (c) and community interventions to improve physical and social environment not directly trageted at people aged 60 years and above.

### 2.5. Information Sources

The searches for articles were made in four databases: CINHAL, EMBASE, PubMed, and PsycINFO. The search targeted only English language studies with full access published between 2008 and 2018.

### 2.6. Key Terms and Article Search

Key terms used for the exposure variables were *Physical activity OR Exercise*. Key terms used for mental health and wellbeing were *Mental health, Mental Disorder, Depression, Anxiety, Wellbeing, Well-being, Well being, Psychological health, Self-esteem, Self esteem, Self-perception, and self perception*. The terms used to locate older participants were *Aging, Ageing, Aged, Frail, Elderly, Senior citizens, Old age, Senior adults, Adult, Older, Elderly, Elder and Geriatric*. Key terms used for the countries were *Southeast Asia, South East Asia, and South Asia. Searches for the countries were also made including the individual names of all the countries in these two regions* (see Supplementary [Supplementary-material supplementary-material-1] for full electronic search strategy in PubMed).

**Figure 1:**
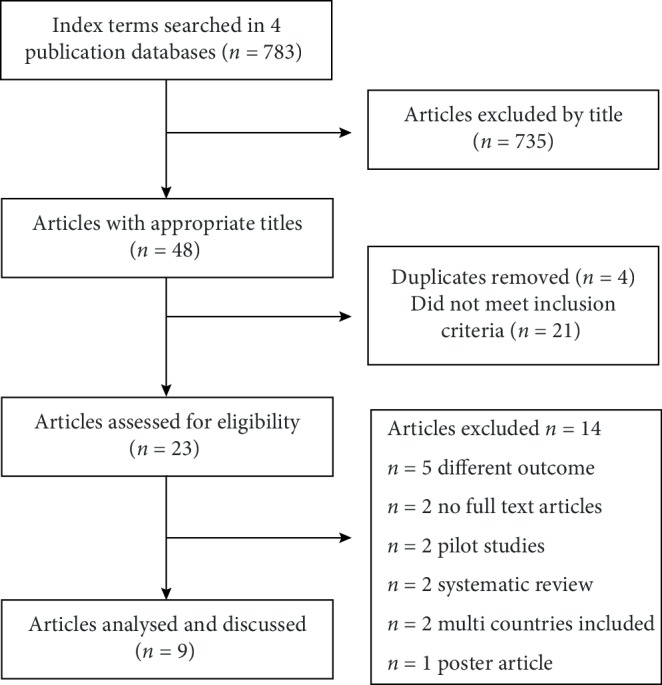
Flow chart showing the selection of studies included in the review.

Selection of sources of evidence: The article selection process is illustrated in Figure 1. At the first stage of selection, SK and RG considered the abstracts for inclusion and exclusion. Only peer-reviewed empirical articles were selected. All articles that met the inclusion criteria were then considered relevant and retrieved for further review. Articles were also identified from the reference lists of articles found during the search process. In the full review, those articles with outcome variables of older adults' mental health and wellbeing measured in relation to physical activity were considered. The articles failing to meet these criteria were rejected. All duplicate articles were removed from the list, and so were the pilot studies, and the studies to which the authors could not get full access. Only those articles that were written in English were selected.

### 2.7. Data Charting Process

We extracted information about each study's design, country of origin, data sources, population characteristics (sample size, age range, population type), intervention details, key findings, and wellbeing measures. We identified the variables that were used to assess mental health and wellbeing of older adults, and also the measures of physical activity and exercises (observations and interventions). Thereafter, we isolated the results for analyses. After that, analysis was conducted by the research team through review and re-review of the findings of the included articles.

### 2.8. Synthesis of Results

The information from the selected studies were organized in tables. The final version of the table has been presented in the results section. We have discussed, compared, and contrasted the findings of different studies in the results section following the table.

## 3. Results

### 3.1. Study Characteristics

Of the 9 articles that were selected for the final analyses, 7 were conducted in Taiwan and 1 each in Pakistan and Thailand as shown in Table 1. The settings of these studies were mostly community (*n* = 8) and only one was facility-based. The study designs adopted in these studies were cross sectional (*n* = 3), quasi experimental (*n* = 3), longitudinal (*n* = 1), and randomized controlled trial (*n* = 2). So, for the purpose of analyses, the studies were divided into two broad categories: the studies involving interventions (*n* = 4) and the studies with no intervention involved (*n* = 5). The sample size ranged between 50 and 1160.

### 3.2. Measurement of Physical Activity as Exposure Variable in the Observational Studies

All five observational studies included in the review involved measuring physical activity using subjective measures (interview or self-reporting questionnaires). Three of the noninterventional studies were cross-sectional [1, 8, 17], while the remaining two were prospective, longitudinal follow-up studies [5, 18]. All five of these studies involved older adults that were community dwelling, i.e., they were not selected from residential care facilities (Table 2).

To quantify physical activity, the respondents were asked about the kinds of activities they were involved in, the total time they were involved in those activities [1, 5, 8, 17, 18] and the intensity of the activities [1, 18]. In studies where intensity was not measured, the physical activity (PA) involvement was measured in terms of weekly frequency (hours/week). However, one study measured both intensity and weekly frequency [4]. Two studies measured physical activity in terms of regularity defined over the course of 6 months [1, 5]. The defining criteria for regularity differed in these two studies in terms of how long each session lasted. The first of these two required only 10 minutes per session [1], while the second required every session to be at least 20-minute long [5].

The types of physical activity assessed included running, mountain climbing, swimming, walking up the stairs, aerobic exercise, fast and normal-speed bicycle riding, jumping ropes, carrying heavy things or babies, shovelling dirt, dancing, yard/house work, playing tennis, playing baseball, and playing table tennis. Likewise, activities also included things done at employment/work or leisure like walking or exercising, shopping, meeting friends, carrying weights of at least 5 kg per week, time spent standing or sitting, and walking during work.

### 3.3. Measurement of Physical Activity as Exposure Variable in the Interventional Studies

As shown in Table 3, two of the four interventional studies adopted a quasi-experimental design and the remaining two studies were cluster-randomized trial and randomized controlled trial.

The interventions adopted for these studies were—the elastic band programme, the silver yoga exercise programme, Tai Chi, and Baduanjin exercise.

The longest intervention lasted for 6 months and the study data were collected at baseline, 3 months, and 6 months in this longest-lasting study out of the four [16]. Other studies were run for 3.5 months [19], 3 months [20], and 6 months [21], respectively. None of the control groups received any intervention and were followed up for their routine activity involvement. Two of these studies involved senior adults visiting activity centers [21, 22], and one each involved senior adults living at residential care facility [19] and home [20].

### 3.4. Variables Used to Quantify Mental Health in the Observational Studies

Four out of five observational studies had depression as their mental health outcome variable [1, 5, 17, 18]. One of those studies however, had mental status as one more outcome variable in addition to depression [5]. The remaining observational study had wellbeing as its outcome variable; however, this variable also comprised depression as one of its six dimensions. Other dimensions included were anxiety, positive wellbeing, self-control, vitality, and general health [8].

Likewise, the Chinese version of the 10-item Center for Epidemiological Studies-Depression Scale (CES-D) [1, 18] and the Geriatric Depression Scale [5, 17] were the two commonest tools used for depression assessment in non-interventional studies. General wellbeing schedule was used by a study that employed wellbeing as its primary outcome variable [8].

### 3.5. Variables Used to Quantify Mental Health in the Interventional Studies

Three interventional studies used sleep quality as their outcome variable and it was measured by using the Chinese version of Pittsburgh Sleep Quality Index (PSQI) [19, 20, 22]. Likewise, in other studies, depression was assessed using the Taiwanese Depression Questionnaire [21], mental health perception was assessed using the mental health component of the Chinese version of SF-12 Health Survey [21], and mental component summary (MCS) derived from the Chinese version of the 12-item Short-form Health Survey (SF-12) was used to quantify self-perceived mental health status [22]. Just like in the study by Lee and Hung, wellbeing of senior adults was also measured using The General Wellbeing Scale (GWBS) in the study by Taboonpong, et al. [8, 20].

### 3.6. Physical Activity Measurement Results in the Observational Studies

The non-interventional studies employed different criteria to determine physical activity among the participants involved. The study by Chang et al. considered each bout of 10 minutes for any activity to count as exercise while the criterion for the study from Pakistan was 20 minutes of activity every day to count as physical activity [1, 17]. In addition to it, the Pakistani study also let the respondents rate their own intensity based on the levels of activity [17].

Walking was the commonest form of physical activity ranging from 43.8% in the Taiwanese adults [5] to (63.4%) in the study among retired elders [8]. Other forms of exercises that were common were Chinese martial arts, swimming, dancing, bicycling, hiking, stretching, tennis, badminton, jogging, and golf [5, 8].

The studies that made comparison across genders found that men were more frequently active [8, 17], spent more time than women on average per week being physically active and exhibited low levels of no activity [5, 17]. Likewise, men were also more likely to have more minutes spent in light activity and vigorous activity (30.8%, 5.4%) than women (25.1%, 5.1%) as demonstrated by the study from Pakistan [17]. In terms of diversity of activities, men tended to be more engaged in walking and hiking while women did more stretching in comparison. Likewise, women perceived hiking and yoga as too intense exercises, whereas men considered aerobics as intense as demonstrated by the study by Wang et al in Taiwan [5].

### 3.7. Mental Health Outcomes with Respect to Physical Activity in the Observational Studies

Those who were regularly physically active had lower odds of having depressive symptoms (OR = 3.54, CI: 1.76–7.12) according to the study in Northern Taiwan [1]. Wang et al. [5] also found that those who were irregular in their exercise were found to have higher depression (*p* < 0.05) compared to those who were regular. Mental status of the regulars and irregulars were also different, with those being regular in exercise more likely to have better mental health status over time (*p* < 0.05). Another Taiwanese study by Lee and Hung also found the exercise frequency to positively impact wellbeing in terms of depression, vitality and positive wellbeing among senior adults [8]. Similarly, the study from Pakistan found that a protection factor of 21.6% against depression could be attributed to spending more than 7 h every week in physical activity alone [17]. Final analyses also demonstrated that higher initial physical activity at baseline was associated with a slower increase in depressive symptoms, among those participants who had not already become depressed at baseline, thus implying future protection [18].

Some findings were, however, more specific in terms of duration and intensity of physical activity and suggested that the mental health-physical activity association might not always be straightforward. For example, Bhamani et al. in the study from Pakistan, pointed out that for the individuals to experience any benefit of being physically active against depression, the senior adults had to be active for more than 5.2 hours in a week. Therefore, those who only spent between 120 and 310 minutes per week in physical activity were not offered significant protection against depression (Adjusted OR = 0.8, 95% CI = 0.4–1.2) [17]. Additionally, according to the study by Chang et al., duration, frequency, and intensity of physical activity had no impact on the respondent's depressive symptoms [1]. Moreover, exercise intensity was even found to have a negative impact on individual wellbeing according to the study by Lee and Hung suggesting that too intense physical activity might do more harm than good to senior adults' mental health [8].

### 3.8. Mental Health Outcomes with respect to Physical Activity in the Interventional Studies

#### 3.8.1. Sleep Quality

Senior adults in the intervention and control groups did not differ significantly in terms of their sleep quality at baseline in any of the three studies that measured sleep quality with respect to physical activity. However, end line comparisons showed that overall sleep quality improved in interventional groups but not in control groups in all three studies.

The study from Thailand did not present the results in terms of subscales of PSQI [20]. However, subscale comparisons between the study with elastic band exercise intervention and the study with the traditional Baduanjin exercise intervention showed mixed results even though the overall analyses showed better sleep quality in intervention groups than in control groups [19, 21].

Both sleep latency and sleep duration improved in the experimental groups compared to the control groups in the studies with elastic band exercise intervention and Baduanjin exercise intervention [19, 21]. Sleep disturbances, daytime dysfunction, and use of sleep medications were not different between interventional and non-interventional groups in the study by Chan and Chen [19]. However, in the study by Chen et al., daytime dysfunction score was significantly worse in the control group as compared with the intervention group [21]. While subjective sleep quality showed significant improvement over the intervention period of 12 weeks in the study by Chen et al. [21], the study by Chan and Chen found that subjective sleep quality of experimental group improved at 3 months compared to the control group [19]; however, this was not maintained at 6 months. The study that used Tai Chi as intervention among older adults showed that the proportion of those with overall better sleep increased postintervention compared to preintervention from 20% to 32% [20]. However, the mean postintervention PSQI in the intervention group was still above the cut-off score of five suggesting overall dismal sleep quality. Noteworthy was also a finding in the study by Chen et al., that the sleep quality in intervention group declined gradually with time, but that for the control group remained unchanged [21].

To summarize, being involved in various forms of physical activity was found to positively contribute to overall better sleep quality among the senior adults as demonstrated by all three studies. However, there was no consistency in the findings for individual subscales.

#### 3.8.2. Depression

The cluster randomized trial that used Silver Yoga exercise as intervention assessed the impact of the intervention on depression. Periodic analyses showed that the intervention group was better than control group in terms of depression state (*F* = 19.14, *p* = .000) after 3 months into the intervention period. Even after six months of intervention, the experimental group was better off than the control group (*p* < 0.05). Therefore, the overall analyses demonstrated that overall depression decreased (*F* = 10.92, *p* = .000) in the intervention group. These findings were in line with the observational studies, which also showed better mental health outcomes, measured in terms of decrease in depressive symptoms, among those who were more physically active than those who were not [1, 5, 17, 18].

#### 3.8.3. Other Outcomes (Mental Health Perception, Mental Component Summary, and Wellbeing)

The other two mental health outcomes showed mixed results. The study using Elastic band exercise intervention and the study using Silver Yoga intervention, with mental health perception as the outcome variable showed non-significant difference, and mental component summary showed no difference between the intervention and control groups at baseline [19, 22]. Three months into the intervention period, the intervention group was better off than the control group (*F* = 5.59, *p* = 0.020) in terms of mental health perception (MHP) [22]. Following six months of intervention, the experimental group was still better off than the control group (*p* < 0.05) [22].

On the contrary, mental component summary had neither any difference in mean scores between the intervention and control groups between baseline and three-month interval (95% CI = −0.73 to 5.05, *p* = 0.143) nor between the baseline and the six-month interval (95% CI = −0.91 to 4.83, *p* = 0.181) as seen in the study by Chan and Chen [19]. Furthermore, the MCS score declined over time in the intervention as well as in the control group. However, the decline was more pronounced in the control group than in the intervention group [19].

As shown by the study employing Tai Chi as the physical activity intervention, the measure of wellbeing increased significantly in experimental group but not in the control group after the intervention. However, the difference in change between the two groups was not statistically significant. Therefore, it was concluded that wellbeing was not significantly improved by the intervention [20].

## 4. Discussion

Alongside understanding the demographic characteristics of senior adults involved in physical activity, and the types of physical activity performed by them in selected countries of South Asia and South East Asia, this study was undertaken also to understand the effects of physical activity on mental health and wellbeing of older senior adults in those countries. The studies that met the inclusion criteria therefore were included in the final analyses were from 3 countries of Taiwan (7), Pakistan (1), and Thailand (1). The mental health outcomes that were assessed in these studies were depression [1, 5, 17, 18, 22], sleep quality [19–22], wellbeing [8, 20], and other measures like self-perceived mental status [5, 22] and mental component summary [19].

### 4.1. Demographic Differences in Physical Activity

It was found that most study participants in these studies fell inside the age bracket of 60 to 75 years. Most of these studies (7 out of 9 were from Taiwan) also demonstrated that these older people were physically active. This observation has also been made in the past in the context of Taiwan [23]. Older men were found to be more frequently active [18], spent more time being involved in a physical activity and tended to be more regular in their physical activity routine [5] compared to older women. This could also be a reflection of a cultural aspect of life in Pakistan [17] and Taiwan [5, 18] where women tend to stay indoors and take care of the housework while men tend to spend more time outdoors. However, when housework was also considered in daily physical activity as in the case of the study from Northern Taiwan, more women than men were found to be engaged in moderately vigorous physical activity [1]. This could also be suggestive of a higher engagement of women in indoor household activities than men. This also suggests that if housework is included in these subjective physical activity measures, women will probably not be seen as lagging behind men in terms of frequency, time spent as well as regularity of physical activity.

Coincidentally, three of the four interventional studies that were included in the review [19, 21, 22] had higher number of women participants than men. It could also be suggestive of higher affinity among women to lighter physical activity than men. These studies included elastic band exercise, silver yoga, and Baduanjin exercise, which were all moderate to light physical exercises. Likewise, as seen in the study by Lee and Hung, older women found hiking and yoga to be very intense, while older men tended to perceive only aerobics as an intense activity. Along the same line, older men practiced walking and hiking more often while older women were more likely to be engaged in stretching [8].

### 4.2. Types of Physical Activity

All of the activities amounting to exercise or physical activity in this study were recorded subjectively. In the observational studies, depending on the operational definition of what comprised physical activity, participants were asked what activities they were engaged in. Walking for pleasure or exercise was the commonest form of light physical activity [1, 5, 8, 17, 18, 22]. Other activities included lifting light weights up to 5 kg, going to meet friends or relatives, gardening, going to purchase personal items [17], any kind of leisure time physical activity [18], hiking, jogging, stretching, aerobics; playing tennis, badminton or golf, and martial arts [8]. Likewise, participants were also found mentioning activities of independent daily living like dressing, eating, using toilet, bathing, and grooming and instrumental activities like using the telephone, light to heavy housework, preparing meals, shopping and outdoor transportation [5]. Likewise, ballroom or folk dancing, Tai Chi, qigong, and morning calisthenics were also some other activities mentioned by the study participants [22]. The interventional studies, on the other hand, introduced activities like elastic band exercise [19], Tai Chi [20], Baduanjin exercise [21], and silver yoga exercise [22].

### 4.3. Physical Activity and Depression

Duration, frequency, and regularity of physical activity were significantly associated with lowered levels of depression as demonstrated by studies by Bhamani et al., Ku et al., and Wang et al. [5, 17, 18]. In Pakistan, duration of physical activity was significantly associated with depression: participants who spent more than 310 minutes per week in physical activity, had 60% less likelihood to become depressed compared to the participants spending less than 120 minutes per week for physical activity [17]. In the 11-year follow-up study conducted in Taiwan, it was seen that the number of sessions of physical activity per week earlier in the study period of 11 years was predictive of reduced risk of depressive symptoms in the future [18]. Likewise, in another Taiwanese study, regular exercisers exhibited significant improvement in depression status, but the irregular exercisers showed regressed depression scores, emphasizing the importance of regular physical activity [5].

Another study from Northern Taiwan also found exercise regularity to be significantly associated with lower levels of depression. However, the study yielded a contradictory finding compared to other studies in terms of duration and frequency of physical activity. The study found that duration and frequency of physical activity had no influence upon depressive symptoms and neither had exercise intensity [1].

There was only one experimental study included in this review, which had depression as the mental health outcome. The Taiwanese study concluded that in a group of older adults who were not statistically different at baseline, the state of depression decreased in the experimental group who was exposed to silver yoga exercise, and it increased in the control group [22].

The positive effect of physical activity on depressive outcomes in senior adults has also been observed in studies across other regions of the world. For example: A 3-year follow-up study among senior adults between 65–75 years in the New York City found that non-disabled senior adults who were athletic, involved in walking or domestic gardening had lower odds of depression [24]. Similarly, intensity of baseline physical exercise was found to be one of the factors influencing future depressive symptoms among senior adults in Finland [25]. Likewise, a study among senior adults in Nottingham, UK also found a modest increase in the risk of depression among those who had lower levels of outdoor or leisure physical activity [26]. Similar was the finding of another study involving women aged 60 years and above in Northern New Jersey, i.e., the higher the levels of physical activity, the lower were the self-reported depressive symptoms. Likewise, in the same study, involvement in a line dancing intervention also led to significantly lower depression among the same group of women [27].

### 4.4. Physical Activity and Sleep Quality

The studies that assessed sleep quality as an outcome measure were all interventional in nature [19–22]. All of these studies unanimously found that sleep quality of the older adults who were in the experimental groups not only improved compared to the baseline but also showed significant positive difference with the sleep quality of older adults in the control groups. It was also found that the interventions started having positive impact on sleep quality within a short interval after the start of the intervention, within 4 weeks in the case of study by Chen et al. and within 3 months in the case of study by Chan and Chen. Those positive changes were found to continue throughout the remaining period of the interventions in both of these studies involving Elastic band exercise and Baduanjin exercise, respectively [19, 21].

Similar findings of better sleep quality among physically active senior adults have been found by studies conducted elsewhere. A systematic review and meta-analysis found that exercise can positively impact sleep quality in older adults [28]. Another meta-analysis found that moderate intensity aerobic exercise or high intensity resistance exercise between 10 and 16 weeks led to moderate beneficial effect on sleep quality as demonstrated by a reduced global Pittsburgh Sleep Quality Index score. Other subdomains of sleep like subjective sleep quality, sleep latency and sleep medication usage were also positively impacted by exercise training [29]. Likewise, another study from Chicago, USA also found that senior adults exposed to 16 weeks of aerobic physical activity and sleep hygiene education had better sleep quality compared to the controls. They also performed better in terms of sleep latency, sleep duration, daytime dysfunction, and sleep efficiency [30]. Another study from China among community-dwelling Chinese older adults found that regardless of factors like age, sex, education, family income, number of children, drinking and sleep hygiene, those who had a greater level of physical activity in comparison, were found to have a better quality of sleep outcome compared to their counterparts [31].

### 4.5. Physical Activity, Wellbeing and Other Mental Health Measures

Another outcome variable employed by two of the reviewed studies was wellbeing. It was used by one observational study by Lee and Hung and one interventional study by Taboonpong et al. The first study found that exercise frequency was positively associated with wellbeing. More frequent bouts of physical activity contributed to better general wellbeing in older adults. As explained above in the section of depression, physical activity frequency and depression had also shown a similar pattern: the higher the frequency, the lower the levels of depression [8, 20].

However, the same study found that high intensity exercise had a negative impact on the older adults' sense of wellbeing. Therefore, it implied that self-assessed low-to-moderate intensity exercise could be good for the psychological wellbeing of older adults when done frequently, but the outcome started shifting to the negative when physical activities got more intense. This finding is intuitive in the sense that older adults are engaged in physical activity in general not to demonstrate their endurance for high intensity physical activity but instead to keep themselves fit for their age and to reinforce their sense of wellbeing. This finding was also supported by similar findings from a meta-analysis where it was seen that longer exercise duration, which is naturally more tiring compared to exercise involving smaller shorter bouts, was less beneficial for the well-being of senior adults [32].

The experimental study involving Tai Chi intervention also found that general wellbeing had significant positive changes in the experimental group compared to the control group. However, even though there was a significant increase in wellbeing in the experimental group compared to the baseline, the difference in the wellbeing measure between the experimental and control group was not statistically significant [20].

The positive impact of physical activity on mental wellbeing as seen in the aforementioned studies was also demonstrated by previous studies. A meta-analysis of 13 studies reported that mental wellbeing in later life could be achieved through physical activity and exercise [33]. Another meta-analysis of 36 studies found that the mean-change effect size in mental wellbeing for physically active senior adults was almost 3 times that for those senior adults who were not physically active. Moderate intensity activity was found to have the most benefit in terms of mental wellbeing [32].

Self-perceived mental health status and mental component summary were also assessed in relation to physical activity in 3 studies [5, 19, 22]. Mental health status either improved or showed less deterioration among regular exercisers compared to irregular exercisers as demonstrated by the study conducted among older Taiwanese adults, reinforcing the importance of regularity in physical activity for positive mental health outcome as also demonstrated for other components like depression and sleep quality [5]. Likewise, in the experimental study using silver yoga exercise as an interventional measure, all the mental health indicators in the experimental group participants were found to be better than in the control group participants [22].

On the other hand, the experimental study, which utilized elastic band exercise as the intervention, found that self-perceived mental health status was not significantly different between the experimental and control groups either at the third month or at the end of interventional period of six months, compared to the baseline. However, it did find that the rate of decline in mental health score was greater in control group compared to the experimental group implying that the intervention did have a positive impact on the experimental group [19]. A significant positive association between physical activity and self-perceived mental health was also observed among senior adults in Czechia [34]. Similarly, Whitehall II cohort analysis among early old age adults from the UK also demonstrated a bidirectional association between physical activity and mental health. Physical activity and mental health improved over time and those with faster improvements in either variable experienced corresponding change in the other variable [35].

### 4.6. Limitations

The review attempted to include as many countries as possible from South Asia and Southeast Asia, but we could only review studies from 3 countries due to other studies not meeting the review eligibility criteria. Therefore, the findings might not apply to all countries from South and Southeast Asia. Likewise, all studies included in the review relied on subjective reporting of physical activity, which might not reflect the real levels of physical activity practiced by older adults. Due to limited countries included in the review, it was not possible to draw region-level comparative analyses. Likewise, the study only included the older adults without any diagnosed illnesses, so the study findings do not represent those older adults with one or more diagnosed illnesses. The study also has a limitation in terms of the time period covered by the review since only the studies conducted between 2008 and 2018 were included.

## 5. Conclusion

With the ever-increasing population of older people in South Asia and Southeast Asia, it is important to understand whether physical activity could be a way to improve the quality of life through positive mental health and wellbeing outcomes in older adults. As found in this review, factors like exercise frequency, regularity, and duration seem to have a positive impact on mental health and wellbeing of older adults. Likewise, any kind of intervention, which got older people to be more active, was found to positively contribute to their mental health and wellbeing as long as it was within a reasonable intensity. More wide-encompassing evidence through studies employing objectively measured physical activity incorporating various forms of physical activity/exercise practiced across different countries and cultures will help better inform public health strategies that can contribute to the positive mental health and wellbeing of older adults.

## Figures and Tables

**Table 1:** Overview of the studies included in the review.

Characteristics	Number of studies (*n* = 9)	Characteristics	Number of studies (*n* = 9)
Country		Study design	
Taiwan	7	Cross-sectional	3
Pakistan	1	Quasi experimental	1
Thailand	1	Longitudinal	2
		Randomized control trail	1
		Cluster randomized control trail	2
Data type		Sample size	
Primary	8	<500	6
Secondary	1	500–1000	1
		>1000	2
Study setting			
Community based	8		
Facility based	1		

**Table 2:** General overview of non-interventional studies (*n* = 5).

S.no.	Title and date	Objective(s) of the study	Population characteristics	Mental health and wellbeing measures	Key findings
(1)	Regular exercise and depressive symptoms in community-dwelling elders in Northern Taiwan [1] 2016	To examine the association between regular exercise and depressive symptoms in community-dwelling older adults in Taiwan.	The study participants were older adults aged 65 years and above, living in northern Taiwan. The community-dwelling older adults were selected using probability-proportional-to-size procedure. One thousand and twenty individuals completed the questionnaires.	Center for epidemiological studies depression scale (CES-D) was used to measure degree of depression symptoms.	Regular exercise was the only factor significantly related to a lack of depressive symptoms, both for male and female older adults.
(2)	Depression and its association with functional status and physical activity in the elderly in Karachi, Pakistan [17] 2015	To determine the functional status and level of physical activty and their association with depression in the elderly population.	The study participants were older adults aged 60 years and above living in Karachi, Pakistan. Nine hundred and fifty three individuals were selected using mult-stage cluster sampling in Karachi, Pakistan.	Depression was assessed by the 15-item geriatric depression scale.	Male older adults were more physically active than female counterparts. Participants spending more than 310 min (>5.2 h) per week in physical activity were 60% less likely to be depressed compared to those who spent less than 120 min (<2 h) per week. Strong association was observed between depression and time spent in physical activity.
(3)	Physical activity and depressive symptoms in older adults: 11-year follow-up [18] 2011	To examine the reciprocal associations between changes in physical activity and depressive symptoms among older adults.	The analyses in this study were based on the data from the Taiwan's health and living status of the elderly survey collected in 1996, 1999, 2003 and 2007. Data representing a cohort of 1160 senior adults aged 67 years and above in 1996 were studies with 11 years of follow-up.	The 10-item Chinese version of original 20-item centre for epidemiologic studies-depression scale was used to assess symptoms of depression.	Levels of physical activity were negatively associated with changes in depressive symptoms (*p* < 0.05) while early depressive symptoms were not related to changes in physical activity (*p* < 0.05)
(4)	The relationship between exercise participation and wellbeing of the retired elderly [8] 2011	To identifiy the relationship between physical exercise and the feelings of wellbeing among retired senior adults.	Quota sampling method was adopted to choose the respondents in this face-to-face survey. Three hundred and fifty two questionnaires were collected from selected parks in Taipei, Taiwan for analysis.	The general wellbeing (GWB) schedule developed by Dupuy was used to measure the wellbeing of the participants.	Exercise frequency had a significant positive effect on wellbeing and three dimensions of depression; positive wellbeing and vitality. A negative correlation was observed between wellbeing and exercise intensity. The older adults felt more comfortable and gained more pleasure psychologically while participating in less intensive exercise.
(5)	The health benefits following regular ongoing exercise lifestyle in independent community-dwelling older Taiwanese adults [5] 2010	To examine the effect of regular ongoing exercise lifestyle on mental and physical health.	One hundred and ninety seven older adults aged 60 years and above were recruited in this prospective longitudinal follow-up study. Measurements were made at baseline and in a 2-year follow-up assessment. The participants in this Taiwanese study were selected from local community centres.	Chinese version of the mini-mental status examination (C-MMSE) and a Chinese version of the geriatric depression scale (C-GDS) were used to assess mental health.	Regular exercise group were less depressed (*p* = 0.03) and tended to regress less on the performance tests (*p*= 0.025–0.410) across 2 years compared to the irregular exercise group.

**Table 3:** General overview of interventional studies (*n* = 4).

S.no.	Title and date	Intervention	Population characteristics	Key findings	Mental wellbeing measure
(1)	Self-perceived health status and sleep quality of older adults living in community after elastic band exercises [22] 2016	The elastic band programme included three stages, warm up, aerobic motion and static stretching. Each phase was executed for 12 minutes, 10 minutes, and 18 minutes respectively. The intervention was conducted 3 times per week with each session lasting for 40 minutes for six months.	The study was conducted in six senior-citizen centers, southern Taiwan. Out of total 199 participants recruited, a total of 169 participants were included for final data analysis where 84 constituted the experimental group and 85 constituted the control group.	At the three month interval, compared to participants in control group, the participants in the experimental group experienced the greater improvemnet in self-perceived physical health, overall sleep quality, sleep latency and sleep duration.	Chinese version of 12-item short -Form Health Survey (SF-12) was used to measure the self-perceived health status of participants including physical and mental aspects.
					Chinese verison of the Pittsburgh Sleep Qualty Index was used to measure sleep quality of the patients.
(2)	Sleep quality, depression state, and health status of older adults after silver yoga exercises: Cluster randomized trial [21] 2009	The silver yoga program included warm up, hatha yoga gentle stretching, relaxation and guided-imagery meditation. Each session was conducted for 70 minutes, three times per week for 6 months.	The study was conducted in 8 randomly selected senior activity centers in Taiwan with total 139 senior citizens,out of which 62 participants from 4 senior activity centers were assigned for experimental goup and 66 to control group randomly.	Silver yoga intervention helped in improving the mental health indicators of the participants significantly in experimental group comapred to control group (all *p* < 0.05). Many of the indicators improved after 3 months of intervention and were maintained throughout the 6 month study.	Taiwanese Depression Questionnaire (TDQ) was used to measure depression state of the participants. Chinese version of SF-12 was used to measure self perception of health status.Pittsburgh Sleep Quality Index (PSQI) was used to measure sleep quality of the patients.
3.	The Effects of Tai Chi on Sleep Quality, Wellbeing and Physical Performances among Older Adults [19] 2008	Tai Chi program comprised of 7 minutes warm up and 15 minutes of 18 basic Tai Chi movements. Each session is 22 minutes, 3 times a week for 12 weeks.	The study was conducted in two similar residential care facilities for older adults in Thailand. Out of which one was assigned as experimental site and other as control site with 25 participants in each.	Experimental group experienced a significant positive changes on the PSQI, GWBS, step test and lung capacity. After pre-post questionnaire comparison, experimental group at 14 weeks had significant reduction of PSQI scòre, which indicated better sleep quality. The change score between two groups of the GWBS, lung capacity and sit and reach test showed no differences.	The Pittsburg Sleep Quality Index (PSQI) was used to measure subjective sleep quality. General wellbeing scale (GWBS) was modified from Dupuy's General wellbeing.
4.	The effect of a simple traditional exercise programme (Baduanjin exercise) on sleep quality of older adults: A randomized controlled trial [20] 2012	The Baduanjin exercise comprised three stages- warm up, exercise and cool down. The Baduanjin exercise program included three parts: a videotape demonstrating Baduanjin exercise, a picture based educational brochure and instructions to perform 30 minutes home based Baduanjin exercise, 3 times a week for 12 weeks.	A total of 202 community dwelling older adults in northern Taiwan were screened for the study; out of which 55 completed the 12 weeks study. Twenty seven participants were randomly assigned to exercise group and 28 to control group.	After 12 weeks of intervention, the experimental group had significant improvement in overall sleep quality, subjective sleep quality, sleep latency, sleep duration, sleep efficiency, and daytime dysfunction (*p* < 0.001)	Chinese version of the PSQI was used to measure sleep quality. The Chinese verison of the short form of the Geriatric Depression Scale (GDS-SF) was used as a screening tool to measure depressive symptoms..

## Supplementary Materials

Supplementary 1 Annex Figure 1: the figure is the spreadsheet matrix/format that was used to enter the information that was extracted from the articles listed for review. Annex Table 1: South Asian and South East Asian countries according to Asian Development Bank (ADB) and South Asian Association for Regional Cooperation (SAARC) classifications. Annex Table 2: Search grid example using the PubMed database.
