# Supplementary material for: Physical Activity, Mental Health, and Wellbeing among Older Adults in South and Southeast Asia: A Scoping Review
Source: Biomed Res Int. 2019 Nov 17;2019:6752182. doi: 10.1155/2019/6752182 (PMC6925721; doi:10.1155/2019/6752182)
Supplement: Supplementary 1 — Annex Figure 1: the figure is the spreadsheet matrix/format that was used to enter the information that was extracted from the articles listed for review. Annex Table 1: South Asian and South East Asian countries according to Asian Development Bank (ADB) and South Asian Association for Regional Cooperation (SAARC) classifications. Annex Table 2: Search grid example using the PubMed database. [file 6752182.f1.pdf]

Preferred Reporting Items for Systematic reviews and Meta-Analyses extension for Scoping Reviews (PRISMA-ScR) Checklist

SECTION	ITEM	PRISMA-ScR CHECKLIST ITEM	REPORTED ON PAGE #
**TITLE**			
Title	1	Identify the report as a scoping review.	Line number 1 and 2
**ABSTRACT**			
Structured summary	2	Provide a structured summary that includes(as applicable): background, objectives, eligibility criteria, sources of evidence, charting methods, results, and conclusions that relate to the review questions and objectives.	Line number 22 to 40
**INTRODUCTION**			
Rationale	3	Describe the rationale for the review in the context of what is already known. Explain why the review questions/objectives lend themselves to a scoping review approach.	Line number 102 to 116
Objectives	4	Provide an explicit statement of the questions and objectives being addressed with reference to their key elements (e.g., population or participants, concepts, and context) or other relevant key elements used to conceptualize the review questions and/or objectives.	Line number 118 to 130
**METHODS**			
Protocol and registration	5	Indicate whether a review protocol exists; state if and where it can be accessed (e.g., a Web address); and if available, provide registration information, including the registration number.	The review protocol was not registered.
Eligibility criteria	6	Specify characteristics of the sources of evidence used as eligibility criteria (e.g., years considered, language, and publication status), and provide a rationale.	Line number 153 to 173
Information sources*	7	Describe all information sourcesin the search (e.g., databases with dates of coverage and contact with authors to identify additional sources), as well as the date the most recent search was executed.	Line number 174 to 177
Search	8	Present the full electronic search strategy for at least 1 database, including any limits used, such that it could be repeated.	Line number 179 to 216
Selection of sources of evidence†	9	State the process for selecting sources of evidence (i.e., screening and eligibility) included in the scoping review.	Line number 179 to 216
Data charting process‡	10	Describe the methods of charting data from the included sources of evidence (e.g., calibrated forms or forms that have been tested by the team before their use, and whether data charting was done independently or in duplicate) and any processes for obtaining and confirming data from investigators.	Line number 218 to 225
Data items	11	List and define all variables for which data were sought and any assumptions and simplifications made.	Excel file is attached showing the data items
Critical appraisal of individual sources of evidence§	12	If done, provide a rationale for conducting a critical appraisal of included sources of evidence; describe the methods used and how this information was used in any data synthesis (if appropriate).	The eligibility criteria guided this process. No separate critical appraisal was done.

SECTION	ITEM	PRISMA-ScR CHECKLIST ITEM	REPORTED ON PAGE #
Synthesis of results	13	Describe the methods of handling and summarizing the data that were charted.	Line number 226 to 229
**RESULTS**			
Selection of sources of evidence	14	Give numbers of sources of evidence screened, assessed for eligibility, and included in the review, with reasons for exclusions at each stage, ideally using a flow diagram.	Flow diagram is presented in the document to show this process.
Characteristics of sources of evidence	15	For each source of evidence, present characteristics for which data were charted and provide the citations.	The characteristics used for data charting were guided by the research questions and related to the column names presented in Excel file.
Critical appraisal within sources of evidence	16	If done, present data on critical appraisal of included sources of evidence (see item 12).	The eligibility criteria guided this process. No separate critical appraisal was done.
Results of individual sources of evidence	17	For each included source of evidence, present the relevant data that were charted that relate to the review questions and objectives.	Excel file is attached showing the names of columns under which data were collected.
Synthesis of results	18	Summarize and/or present the charting results as they relate to the review questions and objectives.	Line number 230 to 408
**DISCUSSION**			
Summary of evidence	19	Summarize the main results (including an overview of concepts, themes, and types of evidence available), link to the review questions and objectives, and consider the relevance to key groups.	Line number 409 to 568
Limitations	20	Discuss the limitations of the scoping review process.	Line number 569 to 580
Conclusions	21	Provide a general interpretation of the results with respect to the review questions and objectives, as well as potential implications and/or next steps.	Line number 581 to 592
**FUNDING**			
Funding	22	Describe sources of funding for the included sources of evidence, as well as sources of funding for the scoping review. Describe the role of the funders of the scoping review.	No funding for the review.

JBI = Joanna Briggs Institute; PRISMA-ScR = Preferred Reporting Items for Systematic reviews and Meta-Analyses extension for Scoping Reviews.

* Where *sources of evidence* (see second footnote) are compiled from, such as bibliographic databases, social media platforms, and Web sites.

† A more inclusive/heterogeneous term used to account for the different types of evidence or data sources (e.g., quantitative and/or qualitative research, expert opinion, and policy documents) that may be eligible in a scoping review as opposed to only studies. This is not to be confused with *information sources* (see first footnote).

‡ The frameworks by Arksey and O'Malley (6) and Levac and colleagues (7) and the JBI guidance (4, 5) refer to the process of data extraction in a scoping review as data charting.

§The process of systematically examining research evidence to assess its validity, results, and relevance before using it to inform a decision. This term is used for items 12 and 19 instead of "risk of bias" (which is more applicable to systematic reviews of interventions) to include and acknowledge the various sources of evidence that may be used in a scoping review (e.g., quantitative and/or qualitative research, expert opinion, and policy document).

From: Tricco AC, Lillie E, Zarin W, O'Brien KK, Colquhoun H, Levac D, et al. PRISMA Extension for Scoping Reviews (PRISMA-ScR): Checklist and Explanation. Ann Intern Med. ;169:467–473. doi: 10.7326/M18-0850

Systematic Literature Search Demonstration

Included countries: South Asian and South East Asian countries according to Asian Development Bank (ADB) and South Asian Association for Regional Cooperation (SAARC) classifications:

Afghanistan	South Asia	Brunei	South East Asia
Bangladesh	South Asia	Burma	South East Asia
Bhutan	South Asia	Cambodia	South East Asia
India	South Asia	Indonesia	South East Asia
Maldives	South Asia	Laos	South East Asia
Nepal	South Asia	Malaysia	South East Asia
Pakistan	South Asia	Philippines	South East Asia
Sri Lanka	South Asia	Singapore	South East Asia
		Thailand	South East Asia
		Vietnam	South East Asia

Databases searched:

Pubmed-Medline, CINAHL, EMBASE, PsycINFO

Search grid example for PubMed

**Database**	**Latest search date**	**Physical activity**	**Senior adults**	**Mental health and well-being**	**South East Asia**	**Combined total hits**
PubMed	05-06-2018	Exercise[mh] OR Physical activity*[tiab] OR Active*[tiab]	Aged[mh] OR Adult*[tiab] OR Frail elderly [tiab] OR Old*[tiab] OR Senior adult*[tiab]	Mental health [mh] OR Mental Disorder*[tiab] OR Depress* [tiab] OR Anxiety[tiab] OR Well-being[tiab] OR Self esteem*[tiab]OR Self perception[tiab]	Asia, Southeastern[mh] OR Asia, Western [mh] OR Afghanistan*[tiab] OR Maldives*[tiab]	
No. of hits		1101837	4539246	606436	322945	211

S.N.	Title	Journal name	Database	Year	Country	Objective	Study design	Population Characteristics	Inclusion and Exclusion criteria	Intervention/ Exposure	Main findings	Mental well being measures	Conclusion	Source URL
